# Probing ligand selectivity in pathogens

**DOI:** 10.7554/eLife.94720

**Published:** 2023-12-21

**Authors:** Bryan VanSchouwen, Giuseppe Melacini

**Affiliations:** 1 Department of Chemistry and Chemical Biology, McMaster University Hamilton Canada; 2 Department of Chemistry and Chemical Biology, and the Department of Biochemistry and Biomedical Sciences, McMaster University Hamilton Canada

**Keywords:** Protein kinase A, ligand selectivity, trypanosomatid pathogens, *T. brucei*, *T. cruzi*, *L. donovani*, Other

## Abstract

Why does protein kinase A respond to purine nucleosides in certain pathogens, but not to the cyclic nucleotides that activate this kinase in most other organisms?

**Related research article** Ober V, Githure GB, Santos YV, Becker S, Moya G, Basquin J, Schwede F, Lorentzen E, Boshart M. 2023. Purine nucleosides replace cAMP in allosteric regulation of PKA in trypanosomatid pathogens. *eLife* **12**:RP91040. doi: 10.7554/eLife.91040.

Protein kinase A (PKA) is involved in a wide range of intracellular processes within eukaryotic organisms, and it also has an important role in the life cycles of a number of pathogens that cause various deadly diseases. These include *Trypanosoma brucei,* which causes sleeping sickness (Bachmaier et al., 2019); *Trypanosoma cruzi,* which causes Chagas disease (Baker et al., 2021); and the parasites that cause leishmaniosis (Cayla et al., 2022). An ability to target PKA in these pathogens – which are collectively known as trypanosomatid pathogens – could lead to new treatments for these diseases, but only if this can be done without affecting PKA in the body of the person with the disease.

Such selective targeting and inhibition may be possible because, in most organisms, PKA is activated by a cyclic nucleotide called cAMP (Rinaldi et al., 2010; Haste et al., 2012; Kurokawa et al., 2011; Taylor et al., 2012; Khamina et al., 2022). However, PKA in *T. brucei* does not respond to cAMP, and is activated instead by purine nucleosides. These nucleosides are similar in structure to cyclic nucleotides, but lack a phosphate group (Figure 1A and B).

**Figure 1.**
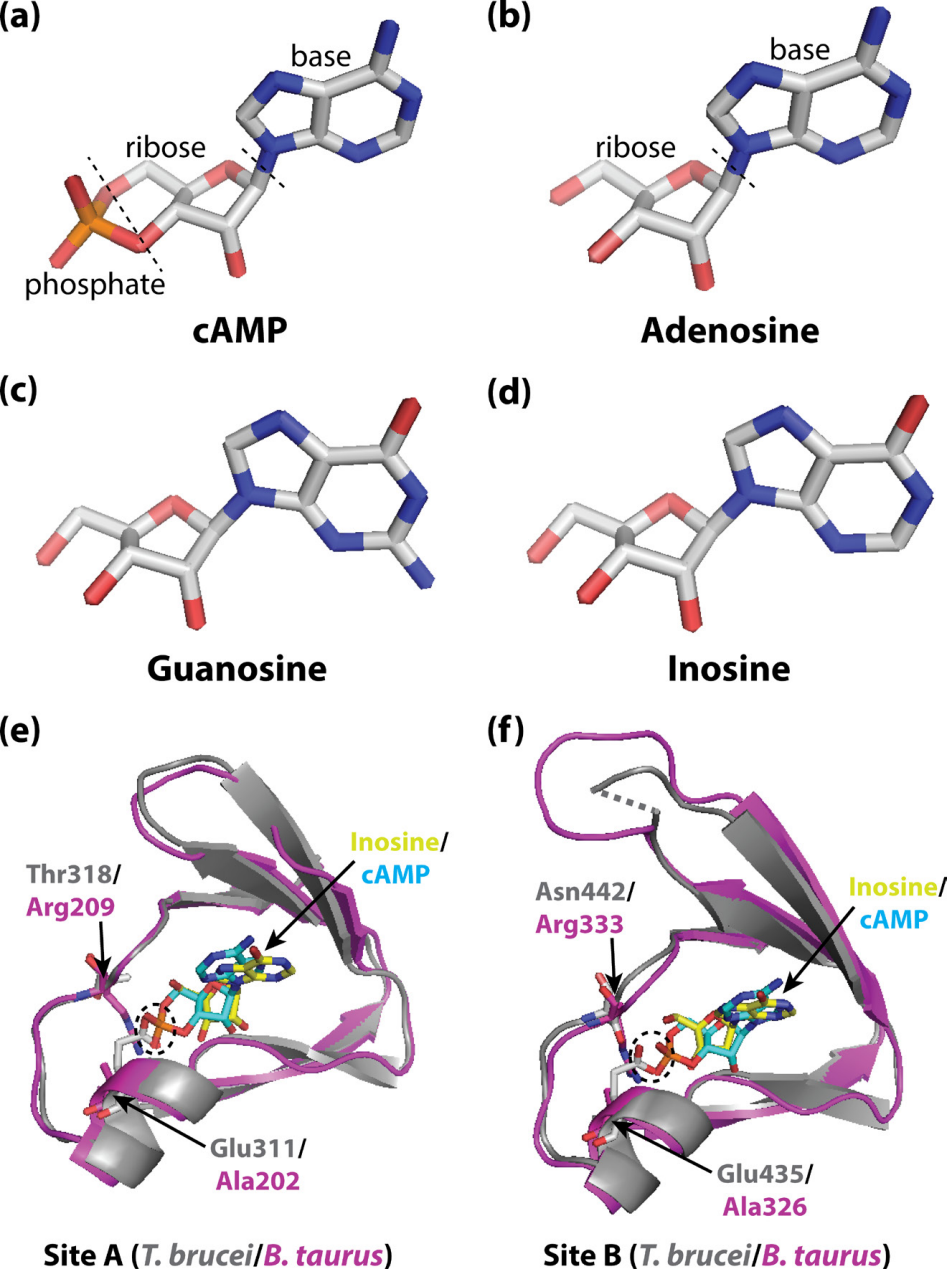
Activating PKA in mammals and trypanosomatid pathogens. (**A–D**) Stick representations of the molecular structures of the cyclic nucleotide cAMP (**A**), and three purine nucleosides: adenosine (**B**), guanosine (**C**) and inosine (**D**). The main difference between these molecules is that cAMP contains a phosphate group which the purine nucleosides lack. In most organisms, an important kinase called PKA is activated when two cAMP molecules bind to two tandem binding sites in the regulatory subunit of the kinase (Su et al., 1995; Kim et al., 2007; Akimoto et al., 2015). In trypanosomatid pathogens, on the other hand, PKA is activated when purine nucleosides bind to these sites. (**E**) Ribbon/stick structures showing a purine nucleoside (inosine; yellow sticks) bound to *T. brucei* PKA at site A (grey ribbons), overlaid with cAMP (cyan sticks) bound to the same site for mammalian (*Bos taurus*) PKA (purple ribbons). (**F**) Ribbon/stick structures showing inosine and cAMP bound to PKA at site B. The amino acids that differ between mammalian and trypanosomatid PKA are labelled: Arg209 and Arg333 in mammalian PKA are replaced by Thr318 and Asn442 in trypanosomatid PKA, while Ala202 and Ala326 are replaced by Glu311 and Glu435. For clarity, other parts of the PKA structures have been omitted. The proposed clash between Glu311/Glu435 of trypanosomatid PKA and the phosphate group of cAMP that prevents cAMP from binding is also indicated (dashed ovals). PKA: protein kinase A; the protein structures used for PKA can be found at PDB ID 6FLO (*T. brucei*) and PDB ID 1RGS (*B. taurus*).

Now, in eLife, Michael Boshart (Ludwig-Maximilians-University Munich) and colleagues – including Veronica Ober, George Githure and Yuri Volpato Santos as joint first authors – report the results of experiments which shed light on the activation of PKA in *T. brucei* and other trypanosomatid pathogens, opening new opportunities to develop novel treatments for the diseases caused by such pathogens (Ober et al., 2023).

To start, Ober et al. assessed structure-activity relationships for kinase activation by various ligands, and found that three purine nucleosides – inosine, guanosine and adenosine (Figure 1B–D) – were direct activators of PKA in three trypanosomatid pathogens (*T. brucei, T. cruzi* and *Leishmania donovani*), with inosine being the most potent of the ligands. Moreover, no activation by cAMP was observed, in agreement with previous findings for *T. brucei* (Bachmaier et al., 2019). The results suggest that activation by purine nucleosides, and insensitivity to cAMP, are universal features of these pathogens.

Consistent with these results, when Ober et al. measured ligand binding affinities for *T. brucei* and *L. donovani* PKA, inosine exhibited the highest affinity, and there was no significant binding of cAMP. For human PKA, on the other hand, cAMP exhibited high-affinity binding, and there was no significant binding of the three nucleosides. Furthermore, analysis of PKA-bound ligands purified from *T. brucei* cells at various stages of their life cycle confirmed the presence of all three nucleosides – inosine, guanosine and adenosine – in vivo.

To explore these interactions in detail, Ober et al. – who are based at institutes across Germany, and also in Denmark – solved three-dimensional crystal structures of inosine bound to the regulatory subunits of PKA from both *T. cruzi* and *T. brucei*. From these structures, they identified key amino acid residues in the binding sites of PKA that interact with inosine, including interactions with the ribose moiety of inosine that were conserved in both structures.

Comparison of these structures with the structure of cAMP bound to mammalian PKA (Su et al., 1995) highlighted two key differences in each binding site: a conserved arginine residue in mammalian PKA (which interacts with the phosphate group of cAMP) is replaced with a polar amino acid in trypanosomatid PKA; and a conserved alanine residue is replaced with a conserved glutamate residue (which interacts with the ribose of the nucleosides; Figure 1E and F). Notably, the structure comparison suggested that the conserved glutamate residue in trypanosomatid PKA would clash with the negatively charged phosphate of bound cAMP, which would explain why cAMP does not bind and activate trypanosomatid PKA. Furthermore, when these residues were mutated to the corresponding residues found in mammalian PKA, cAMP was able to activate the trypanosomatid PKA.

The work of Ober et al. provides valuable insights into the activation of trypanosomatid PKA at the molecular level. In the future, it would be interesting to explore the complex formed by the regulatory and catalytic subunits of trypanosomatid PKA, and its nucleoside binding affinities, as this complex constitutes the resting state of PKA in other organisms (Kim et al., 2007). Furthermore, there remains the question of how the activation of trypanosomatid PKA by nucleosides is terminated in vivo, as the mechanism responsible is likely different from that found in human PKA (which relies on enzymes called phosphodiesterases).

The more we learn about the differences between trypanosomatid PKA and mammalian PKA, the better chances we have of being able to selectively target trypanosomatid PKA in medical treatments for sleeping sickness and other diseases caused by trypanosomatid pathogens.
